# Parental Experiences of the Pediatric Day Surgery Pathway and the Needs for a Digital Gaming Solution: Qualitative Study

**DOI:** 10.2196/23626

**Published:** 2020-11-13

**Authors:** Arja Rantala, Miia M Jansson, Otto Helve, Pekka Lahdenne, Minna Pikkarainen, Tarja Pölkki

**Affiliations:** 1 Research Group of Medical Imaging, Physics and Technology Research Unit of Nursing Science and Health Management, Faculty of Medicine University of Oulu Oulu Finland; 2 Research Group of Medical Imaging, Physics and Technology University of Oulu Oulu Finland; 3 Pediatric Research Center Department of Pediatrics, Helsinki University Hospital University of Helsinki Helsinki Finland; 4 Department of Pediatrics Helsinki University Hospital Helsinki Finland; 5 Research Group of Medical Imaging, Physics and Technology VTT Technical Research Centre of Finland University of Oulu Oulu Finland; 6 Research Unit of Nursing Science and Health Management, Faculty of Medicine University of Oulu Oulu Finland; 7 Department of Children and Women Oulu University Hospital Oulu Finland; 8 Medical Research Center Oulu Oulu Finland

**Keywords:** anxiety, children, day surgery, delivery of health care, digital solution, gamification, nursing, pain, qualitative study, technology

## Abstract

**Background:**

The parents of hospitalized children are often dissatisfied with waiting times, fasting, discharge criteria, postoperative pain relief, and postoperative guidance. Parents’ experiences help care providers to provide effective, family-centered care that responds to parents’ needs throughout the day surgery pathway.

**Objective:**

The objective of our study was to describe parental experiences of the pediatric day surgery pathway and the needs for a digital gaming solution in order to facilitate the digitalization of these pathways.

**Methods:**

This was a descriptive qualitative study. The participants (N=31) were parents whose children were admitted to the hospital for the day surgical treatments or magnetic resonance imaging. The data were collected through an unstructured, open-ended questionnaire; an inductive content analysis was conducted to analyze the qualitative data. Reporting of the study findings adheres to the Consolidated Criteria for Reporting Qualitative Research (COREQ) checklist.

**Results:**

Parental experiences of the children’s day surgery pathway included 3 main categories: (1) needs for parental guidance, (2) needs for support, and (3) child involved in his or her own pathway (eg, consideration of an individual child and preparation of child for treatment). The needs for a digital gaming solution were identified as 1 main category—the digital gaming solution for children and families to support care. This main category included 3 upper categories: (1) preparing children and families for the day surgery via the solution, (2) gamification in the solution, and (3) connecting people through the solution.

**Conclusions:**

Parents need guidance and support for their children’s day surgery care pathways. A digital gaming solution may be a relevant tool to support communication and to provide information on day surgeries. Families are ready for and are open to digital gaming solutions that provide support and guidance and engage children in the day surgery pathways.

## Introduction

### Background

The Global Observatory for eHealth has defined mobile health (mHealth) solutions as “medical and public health practice supported by mobile devices, such as mobile phones, patient-monitoring devices, personal digital assistants (PDAs), and other wireless devices” [1]. These digital or connected health services or solutions are changing medical and public health practices [2]. However, assessment frameworks should respect the needs and capacity of each medical system or country [3]. Gaming and gamification are areas of mHealth, which can expand users’ acquiescence with health interventions and improve users’ capability to self-administer and adherence to treatment [4]. Among health care professionals, developing games should be evidence-based and targeted to those who need them [5]. Gamification includes game design elements in a nongame context [6]. Game elements include competition under rules, a narrative context, a feedback and communication system, and time pressure. In addition, gamification engages users with unparalleled intensity and as per a duration system [6,7]. Serious health games (SHGs) can include educational and physiological elements as well as additional knowledge on how to influence the goal achieved through the game, such as alleviating pain or anxiety among children or parents [5,8].

For children, day surgery procedures are more common than inpatient procedures [9]. Every year, approximately 3%-10% of children (age <17 years) experience hospital stays in developed economies, such as the United States and Europe [9-11]. Day surgery patients are admitted, operated on, and discharged on the same calendar day [12,13]. A day surgical pathway should be based on a well-planned protocol, defining all the steps from planned treatment, to hospital admission, and to hospital discharge within the same day. A day surgical pathway is cost-effective and includes a nurse‐delivered preanesthetic assessment consisting of preoperative education and appropriate postoperative care guidance. Such careful patient guidance reduces hospital readmissions. [14]. Surgical conditions constitute a significant proportion of the global burden of diseases [15]. However, careful patient selection, refinements in surgical and anesthetic techniques and devices, and successful patient outcomes have contributed to the optimal utilization of surgical processes along with early discharge [14].

Outpatient care also includes patients who are sedated for magnetic resonance imaging (MRI) and are discharged after treatment [16]. Guidelines from the Association of Anaesthetists and the British Association of Day Surgery underline the importance of instructions concerning day surgery and its flow [14]. An ideal day surgery pathway includes minimized waiting times, information on day surgery procedures, routine preoperative checks on the day of admission, analgesia and other postoperative information at discharge, and eventually, instructions for telephone follow-up [13]. The information provided should be of high quality, procedure-specific (for families), and age-appropriate (for children) [14,17]. Patients should be admitted to the day surgery units as close as possible to the time of their surgery.

This paper focuses on parental experiences from the pediatric day surgery pathway. Our study is part of the Icory (Intelligent Customer-driven Solution for Orthopaedic and Paediatric Surgery Care) project in which the ecosystem of hospitals, researchers, and technology providers, together with children and families, are codeveloping a digital gaming solution for the day surgery path.

According to the previous meta-analysis, digital gaming solutions can reduce children’s preoperative anxiety and increase parental satisfaction [18]. In addition, digital gaming solutions can be considered as nonpharmacological distraction tools for children. Parents’ experiences may help care providers to deliver more effective, family-centered care that responds to parents’ needs throughout the day surgery pathway [18]. Family-centered care considers the individual needs of parents and children [19] and emphasizes the role of written information intended to reduce parental anxiety and stress regarding day surgeries [20,21]. In the child-centered care, a child is a person with their own voice and joint participation and partnership in a care environment and is considered competent [19].

In day surgery processes, appropriate preoperative preparation is crucial [22,23]. Usually, the day surgical treatment is a unique experience for both the child and family. The incidence of preoperative anxiety in children varies between 40% and 75% [24,25]. In order to avoid unexpected stress, the whole family needs to be prepared for upcoming surgery [26]. Untreated anxiety is associated with increased intensity of pain afterwards [26-28]. In addition, parental anxiety has an enormous effect on children’s preoperative anxiety, which correlates with increased postoperative pain in children [28]. Correspondingly, the factor of postoperative pain is associated with parental satisfaction in pediatric day surgery [29].

The parents of hospitalized children perceive high levels of stress and anxiety [30,31]. In addition, they are dissatisfied with waiting times [32,33], fasting [32], discharge criteria [13,34], and postoperative pain relief [29]. Parental satisfaction with treatment and the care itself, however, is good [29].

Our study focuses on the gap between the information provided to parents and their needs. In addition, the expectations from a digital gaming solution were addressed to support the digitalization of pediatric day surgery pathways. The research questions were as follows:

1. What are the experiences of parents of care in the pediatric day surgery pathway?
2. What are the needs of parents for a digital gaming solution in the pediatric day surgery pathway?

## Methods

### Design

This was a descriptive qualitative study based on the experiences of parents of hospitalized children [35,36].

### Participants and Settings

Participants were asked to volunteer and participate in the study using convenience sampling [35]. The inclusion criteria were as follows: parent or custodian of a child who was receiving a day surgical treatment at the selected hospital, ability to understand and write in Finnish, and access to a laptop or a mobile app for answering the questionnaire. The selected participants were acquainted with the research topic [37] because of the experience of their child’s day surgery pathway in the hospital.

The respondents consisted of parents (N=31) whose children were admitted to otolaryngologic surgery (n=7), plastic surgery (n=3), oral/dental surgery (n=1), ophthalmic surgery (n=1), orthopedic surgery (n=7), soft tissue surgery (n=3), gastroenterological surgery (n=5), vascular surgery (n=1), and MRI (n=3). Out of the 31 parents, 23 (74%) were females aged 30-39 years, and 8 (26%) were males.

The study was conducted at the university hospital in Finland, a 140-bed tertiary care pediatric hospital. The hospital provides specialized health care in pediatrics, including pediatric surgery, child neurology, and child psychiatry. In addition, the hospital has been assigned the national treatment responsibilities in specific pediatric conditions, for example, cardiac surgery and organ transplant. In 2019, a total of 6883 surgical operations were performed of which 3300 were day surgery procedures.

### Data Collection

The data were collected using an unstructured questionnaire. The questionnaire was planned and designed via remote meetings in 2018 by a panel of 7 specialists from the University of Oulu, VTT Technical Research Centre of Finland, Helsinki University Hospital, and Oulu University Hospital. The panel included pediatricians, nurses, and researchers. The questionnaire was designed to gather information on current pathways of children’s day surgeries in order to develop a more digitized pathway; it was based on the findings from an earlier study [2]. The questionnaire was designed to address the research questions using 3 demographic and 6 open-ended questions (Textbox 1). The questionnaire was tested in March 2019 by a PhD student and a professor in the selected hospital with parents who were waiting for treatment for their children. The parents were invited to respond to Questback Essentials’ web-based unstructured questionnaire via a quick response (QR) code using their own mobile phones. A total of 5 parents responded, and thus the questionnaire was considered usable.

**Unstructured questionnaire for parents.**

1. Respondents’ demographics
2. Respondents’ demographics
3. Has your child been in a day surgery unit? Yes/No
4. What kind of treatment has your child had?
5. What kind of information did you receive from the hospital staff about what is going to happen during your child’s day surgery treatment?
6. Could you describe what kind of support you received during your child’s treatment?
7. Can you tell us how your child was involved in the care path provided by the hospital staff?
8. Did you have an access to any treatment-related games during your child’s care that you would have played with your children?
9. Demo of the Icory solution: What do you think of this solution? What kind of solution should be available?

Data collection was conducted from October 2019 to December 2019. The research nurse recruited voluntary respondents from the hospital’s recovery room while children were in the postanesthesia care unit. The parents answered the anonymous questionnaire via their own smartphones or laptops. If the respondent did not have a smartphone or a laptop, one was provided to them by the research nurse. The researchers were not able to identify individual respondents.

### Data Analysis

The qualitative data were analyzed by conducting an inductive content analysis, which included 3 main phases: preparation, organization, and reporting [35,36]. Inductive content analysis is used when knowledge is fragmented or when there is little knowledge of the phenomenon [38]. Content analysis is a method of analyzing written, verbal, or visual communication that distills words into content-related categories for providing new knowledge of the phenomenon [38,39]. The data were analyzed using NVivo 12 (QRS International), a qualitative research software. Demographic data were reported using frequencies and percentages.

First, the data were evaluated for quality by 3 researchers (AR, MMJ, and TP). The data were transferred to NVivo 12. In the analysis, initial impressions were written down as notes. Second, the data were abstracted into open codes and transferred into tables. Similarly, open codes were grouped into categories [38]. Third, those categories were named using a word that was characteristic of the content and were formed into subcategories. Similar subcategories were grouped together and called upper categories. The process produced 294 open codes, 21 subcategories, 9 upper categories, and 4 main categories. The data analysis was conducted by 2 researchers (AR and MMJ), discussed and agreed upon by 3 researchers (AR, MMJ, and TP), and commented on by the researchers who developed the questionnaire (OH, MP, and PL).

### Rigor

Rigor was ensured using the criteria of credibility, dependability, confirmability, transferability, and authenticity [35,40,41]. Credibility and dependability in this study were ensured by designing the questionnaire based on early studies [2,25,42] and based on the actual needs of the selected hospital. Confirmability was established in the analysis process through researcher triangulation (AR, MMJ, and TP). The preparation phase included defining participant inclusion criteria, planning the data collection in questionnaire format to reach data saturation, and selecting the unit of analysis.

Saturation was achieved when the written answers began to repeat themselves in the collected data. Transferability was established in this study by describing the hospital environment in which the study was conducted in order to enable the interpretation and transfer of the results in other contexts. Authenticity was ensured through quotations to indicate the richness of data [41]. The reporting was performed systematically according to the COREQ (Consolidated Criteria for Reporting Qualitative Research) criteria (Multimedia Appendix 1) [43].

### Ethical Considerations

The study was reviewed by the local ethical committee (decision #3181-2018) and was granted a research permit (decision #284-2019). The aim of this study was verbally explained to the respondents by the research nurse, and they were informed that responding to the questionnaire was entirely voluntary. The questionnaire was designed so that it would be impossible to identify the respondents. The study followed the Helsinki Declaration [44]. All participants were informed about the voluntary nature of the research [21,45].

## Results

Analysis of the data for the first research question revealed 3 main categories related to the parents’ experiences of their children’s day surgery pathways: (1) needs for parental guidance (which included 2 upper categories—content of information and patient flow during the day surgery pathway), (2) needs for support in the children’s day surgery pathway (which included the upper categories—physiological support for children and psychological support for children and families), and (3) child involvement in their own pathway (which included upper categories—consideration of an individual child and preparing a child for treatment) (Table 1).

**Table 1:** Parental experiences of the care in the children’s day surgery pathway.

Categories			Experiences
**Main category 1: Needs for parental guidance**			
	**Upper category 1: Content of information**		
		Subcategory 1: Transparency of the pathway	Lack of knowledge regarding operating theater activities Unexpected follow-up overnight Unexpected changes in day surgery pathway Lack of knowledge regarding day surgery pathway Lack of instruction regarding surgical wound Instructions could be sent by email Instructions in the information letter should be updated
		Subcategory 2: Analgesia and amnesia	Lack of knowledge regarding pain management Need for pain management guidance Need for information about anesthesia
	**Upper category 2: Patient flow during the day surgery pathway**		
		Subcategory 1: Physical environment	Lack of information regarding free parking Guidance signs are needed Timely permission for entering the waiting room
		Subcategory 2: Waiting time	The overall waiting time was too long Waiting is challenging with hungry children The waiting time for the operation was too long Progress regarding the waiting time should be shared by nurse Unsustainable schedules
		Subcategory 3: Roles and responsibilities	Doctor did not call following operation Discrepancies in responsibilities Superficiality of information Nurses are better at sharing information
**Main category 2: Needs for support**			
	**Upper category 1: Physiological support for children**		
		Subcategory 1: Eating after operation	Meal requirements Unsuitable food after operation Conflicting information regarding available meals
		Subcategory 2: Environment safety	Hearing protectors for sound-sensitivity
	**Upper category 2: Psychological support for children and family**		
		Subcategory 1: Rewards and trophies	Rewards are pleasing
		Subcategory 2: Psychological needs	Parents’ psychological needs should be enquired about Parental well-being should be considered
		Subcategory 3: Timing of guidance	Correct timing for nursing guidance Parental role and timing in sudden situation
**Main category 3: Child’s involvement in his or her own pathway**			
	**Upper category 1: Consideration of individual children**		
		Subcategory 1: Individual needs	Needs of sound-sensitive children Own devices for sound-sensitive child
		Subcategory 2: Giving more time to children	More time should be given to children by the nurse Calming should be ensured before operation
	**Upper category 2: Preparation for treatment**		
		Subcategory 1: Preparation for treatment	Familiarization with treatment should be ensured
		Subcategory 2: Preparation with games and pictures	Pictures to help preparation Games to help preparation

Analysis of the data for the second research question revealed 1 main category, a digital gaming solution for children and families to support care. This category included 3 upper categories: preparing children and families for the day surgery via the solution, gamification in the solution, and connecting people through the solution (Table 2).

**Table 2:** Parental needs for a gamification solution in the day surgery pathway.

Digital gaming solution for children and families to support care		Experiences
**Upper category 1: Preparing children and families for the day surgery via the solution**		
	Subcategory 1: Preparing via digital gaming solution	General information about surgery Instructions available beforehand in solution
	Subcategory 2: Virtual familiarization with the care environment	Virtual tour Familiarization with operation room beforehand Visibility of real environment
	Subcategory 3: Waiting time in solution	Information on waiting time Distraction from waiting
**Upper category 2: Gamification in the solution**		
	Subcategory 1: Gamification to overcome hospital anxiety and fear	Games with music Videos in solution Fun in order to relieve fear Games for reducing fear
	Subcategory 2: Gamification in support of care	Games for preparation Games for rehabilitation Age-appropriate material for children
**Upper category 3: Connecting people through the solution**		
	Subcategory 1: Interaction between the medical staff, families, and children	Interaction between nurses and patient Support and guidance from staff (apart from game
	Subcategory 2: Peer support	Children telling stories about treatment via solution Ability to share feelings

### Needs for Parental Guidance

The parents described their experiences and ideas regarding the information that could help both families and children address the challenges they experienced in the day surgery pathway. Identified categories were related to the content of information and patient flow during the day surgery pathway.

#### Content of Information

This upper category included 2 subcategories: transparency of the pathway and analgesia and amnesia. Generally, the content of the provided information (eg, admission instructions, dining, fasting, overnight stay, pain management, parking, patient flow—including preoperative preparation, time, and place) was considered sufficient. For instance, one parent wrote:

I think we got proper information throughout the whole treatment process. They told us where to go and how to prepare for the treatment.

However, some respondents faced challenges regarding the transparency of the pathway and analgesia and amnesia, which are subcategories of this upper category.

Transparency of the pathway included the needs for knowledge of information concerning the children’s day surgical pathway. Parents faced challenges in receiving information on the stages, milestones, and procedures of day surgeries. According to parents, there was a lack of knowledge about the discharge criteria. Moreover, sudden changes brought further challenges in obtaining information. For instance, one respondent wrote: “It was supposed to be a day surgery treatment, but our child needed to be monitored overnight.” Correspondingly, respondents made suggestions related to the information on postsurgery care: “I would like to have better instructions on what to be aware of and what not to do with a surgical wound.”

According to parents, a digital solution with different kinds of features (eg, email) could be utilized in order to enhance information transfer, as one of the respondents remarked, “Yeah, an email could have been a working solution” for providing information about the day surgery*.* The implementation of information transfer (eg, individual counseling delivered via face-to-face contact and telephone; written counseling delivered via letter, email, and SMS reminders) was considered sufficient. One of the parents expressed the following view: “The flow of the care pathway was well communicated by the medical staff on the day, before the treatment, and before and after the treatment in the recovery room.”

Analgesia and amnesia included needs for information concerning pain management. According to respondents, there was a lack of information on the management of postoperative pain and treatment-related pain. One of the respondents maintained, “We don’t know anything about that (pain management at home) yet, I have asked about possible pain but...” In addition, there is a lack of knowledge related to amnesia/sedation. For instance, one respondent wrote:

The one thing we were worried about was how amnesia would go and we asked about it. However, that was confirmed in the operation room.

#### Patient Flow During the Day Surgery Pathway

The parents described their experiences and ideas regarding the patient flow in the day surgery pathway. Identified categories were related to the physical environment, waiting time, and roles and responsibilities, which are 3 subcategories of this upper category.

Physical environment was related to the lack of information regarding free parking and guidance signs. The respondents faced challenges in accessing parking and parents’ waiting areas. According to the respondents, more information about the free car parking and waiting places needed to be added to the information letter. One of the respondents described the following challenge:

We haven't been to the place (hospital) before. I verified the location of the car park from the website.

The respondent also added: “The only thing was that the request for entry to the parents’ living room was made too late.”

Waiting time was related to the main challenges and needs. The arrival time, for instance, was considered to be the same as surgery time. One of the respondents expressed this view by stating, “We had time for the treatment but still we had to wait with other parents for 2-3 hours.” Overall, the waiting time was considered too long, and more information regarding the remaining waiting time was warranted. The following excerpt from one of the respondents expresses such a view: “There should have been some kind of information about how long our waiting time (for the treatment) would be.” The timing of patient counseling was also considered nonoptimal in certain circumstances.

Roles and responsibilities were related to the challenges faced by children and parents in their interactions with the hospital staff. It was unclear how and who would announce what. Despite certain promises, surgeons did not share information about the surgery before and after surgery, or they shared information about surgery superficially and briefly. For instance, one respondent wrote, “There was information on the screen in the waiting room that the doctor would call after the treatment, but this never happened*.*”

In addition, the answers received were somewhat indicative/suggestive. However, the respondents made some suggestions regarding the digital gaming solutions and child involvement in their own care pathways.

### Needs for Support

#### Support for Children and Family

The second main category included 2 upper categories that parents described as needs for the day surgical pathway: physiological support for children and psychological support for children and families. The received support (eg, explanations, parents’ involvement, support from nearby, and friendliness of staff) was considered sufficient. In addition, most of the respondents felt that the service provided by the hospital was friendly, attentive, and informative.

#### Physiological Support

This upper category was related to the challenges of eating after an operation and environmental safety. Conflicting information was observed related to postsurgery meals. The postoperation meal requirements were referenced in many responses, as was the need for hearing protectors for sound-sensitive children.

#### Psychological Support for Children and Families

This upper category addressed the need for rewarding children after operation, timing of guidance from nurses in sudden situations, parental role and timing in sudden situations, and lack of psychological support for parents. Parental involvement was also related to the challenges in their roles. One respondent stated, “It was hard in the operating room (before anesthesia), when you should be focused on your child’s excitement and at the same time matters relating to anaesthesia.”

One of the respondents felt that their psychological needs were ignored in the hospital and mentioned, “Psychological needs were not taken into account and they (hospital staff) could have asked us about it*.*”

### Child Involvement in His or Her Own Care Pathway

#### Consideration and Preparation

The parents described their experiences and ideas regarding their children’s involvement in their own care pathways. The identified categories were related to the consideration of an individual child and preparation for treatment, reflecting 2 upper categories in this main category.

Children’s involvement in their own care pathway (eg, answering the child’s questions, talking to the child, turning attention elsewhere, considering the child’s fear, encouraging and praising the child, giving time to situations faced by children, listening to the child, involving the child in the care path, and taking the individual into account) was considered sufficient. For instance, one respondent stated, “My child was also allowed to ask questions that bothered him, and they were answered really well.”

However, some respondents had faced challenges regarding consideration of an individual child and preparing a child for treatment, which were subcategories for this main category.

#### Consideration of an Individual Child

This category was related to the needs of children with special needs and allowing time for children to calm down. The individual needs of sound-sensitive children should be taken into account.

#### Preparation for Treatment

This category included the challenges related to children’s needs for parents in the operation room before anesthesia, needs to familiarize themselves with the hospital environment in advance, and needs of requiring more time to deal with frightening situations. Respondents collectively described these needs as giving time to children before their operations. One respondent explained this need as follows:

Going to the operating room caused a little extra stress, as the speed was faster there than the speed in the restroom. It would have been good to calm the child down a bit before starting the operation.

### A Digital Gaming Solution for Children and Families to Support Care

#### Preparation, Gamification and Connection

Respondents described their needs for a digital gaming solution that could help children and families in children’s day surgery care. This included 3 upper categories: preparing children and families for the day surgery, gamification in solutions, and connecting people through the solution (Table 2).

Games are not implemented in the hospital’s current pathways of pediatric day surgeries. However, parents reported positive attitudes toward a digital gaming solution for pediatric day surgery. For instance, one respondent noted, “Today’s kids are born at this time of technology, so I think games could work well for kids of a certain age.”

In addition, attitudes toward rehabilitation through playing and gaming were positive. The identified requirements for digital gaming solutions were divided into 7 subcategories.

#### Preparing Children and Families for the Day Surgery via the Solution

The proposed needs for a digital gaming solution were related to 3 different items: preparing via a digital gaming solution, virtual familiarization with the care environment, and managing the waiting time with a solution. These are the subcategories of this upper category.

##### Preparing via a Digital Gaming Solution

This subcategory included aspects that could enable children to prepare for their treatment. The solution should include general information about surgery, whereas instructions concerning treatment were supposed to be already available. Information storage in the game would decrease the need for information retrieval. According to respondents, the developed digital gaming solution should be easy to use*.* An informative gaming solution would reduce the need for Googling, as a respondent added, “That kind of solution would reduce need for Google.”

##### Virtual Familiarization With the Care Environment

Virtual familiarization with the care environment included parental needs for a virtual tour for children and families, information about the operating room via virtual visits, and the ability to see the hospital via the solution. In addition, a digital gaming solution could include genuine pictures of various hospital spaces.

##### Managing the Waiting Time With a Solution

This subcategory, managing the waiting time with a solution, was seen as an important requirement for the solution. The solution would need to provide parents with information about the waiting times following the treatments. In addition, the solution could be applied in other circumstances to relieve waiting.

#### Gamification in the Solution

The upper category of gamification in the solution included the following 2 subcategories: gamification to overcome hospital anxiety and fear and gamification in support of care. This upper category also considers parents’ expectations from gamification in the solution.

##### Gamification to Overcome Hospital Anxiety and Fear

This category included parental needs for solutions for children. According to parents, a digital gaming solution could include all sorts of fun and interactive features (eg, videos, games, and music) to reduce fear. The following excerpt expresses this view:

After all, children can’t help but like everything interactive and cool. Even if the device offers nothing but fun for the child, it will certainly be helpful to relieve fear.

##### Gamification in Support of Care

According to respondents, the gamification could include age-appropriate information regarding the most common types of surgeries. According to parents, certain games could be utilized for the preparation for surgery as well as postoperative recovery (eg, rehabilitation). For instance, one respondent stated, “It would be good to prepare themselves for treatment via a solution.”

#### Connecting People Through a Solution

This upper category included 2 subcategories—interactions between medical staff, families, and children and peer support. The parents stated that the digital gaming solution could enable interaction between the hospital and families and enable the peer support: “I like the thought that with the help of the solution parents could connect with nurses.”

According to parents, a digital gaming solution could include children’s own positive stories regarding their day surgery pathways. In addition, a digital gaming solution could enable the sharing of feelings with others in order to reduce fear. One of the respondents expressed, “Being able to share their feelings with others going through similar measures could be a good way to address their fears.”

## Discussion

### Principal Results

To the best of our knowledge, this is the first qualitative study that explores parental experiences throughout the entire pathway of pediatric day surgeries in order to support the digitalization of that pathway in the selected hospital. Our findings revealed that although the current content and information transfer were considered sufficient, parents expected (1) better guidance related to the content of information, (2) more psychological support, and (3) involvement of children in their own care pathway. Additionally, it was found that there was a need for a digital gaming solution that would provide the required information and help families to be better prepared for their oncoming treatments.

### Strengths and Limitations

The results present the experiences of the needs of parents in their children’s day surgery pathways. Almost all (28/31, 90%) children had been in a day surgery, whereas 10% (3/31) respondents had undergone an MRI. The number of respondents was reasonably small—only 31—and the text material they produced (that was analyzed in this study) was brief, as it usually is when responding via the internet. However, the respondents had a fresh perspective on the care the hospital provided to their children, which strengthens our results.

The data could have had a greater breadth if there had been an opportunity to conduct individual or focus group interviews. Then the interviewees could have provided richer material to be analyzed, or different perspectives could have been clarified. However, the saturation was achieved, and the respondents produced texts that included rich material for our inductive content analysis. For future studies, the perspectives of children should be included for broadening and strengthening the results.

It was not possible to get feedback on the results at an organized event at the hospital because the respondents were anonymized, and they did not ask the research nurse any further questions while responding to the anonymous questionnaire. The results are transferable to similar contexts where a hospital has developed its own digital environment, but the generalizability of the results would require further quantitative research with a larger sample size.

### Comparison With Prior Work

The need for support is in line with previous studies [21,23,29], which explained a situation in which parents were unfamiliar with how to conduct postoperative pain management for their children. However, in our study, the parents were ready to view a digital gaming solution as a relevant tool for supporting care in different situations concerning the children’s day surgery pathways. In recent studies, parents also needed more guidance regarding fasting [23,32], equipment used in the operating and recovery rooms [21], discharge criteria [13,34], and postoperative complications [23,29]. In our study, parents wanted to increase gamification, as it was considered as an important aspect of the required guidance. The need for psychological support is in line with previous literature, in that participants were most frequently dissatisfied with waiting times [21,32,33,46]. Thus, interventions aimed at reducing waiting times and raising patient satisfaction are warranted. In our study, parents were ready for a digital gaming solution, which could be used in different kinds of situations, such as waiting for treatment, reducing children’s anxiety, and patient guidance. For future studies or for developing SHGs for parents and children in the day surgery pathway, our study has addressed the first stage of developing a gaming solution. We have identified a target audience and expected outcomes [5].

According to respondents, the digital gaming solution could be used to help families be better prepared for the coming treatments. This could include a virtual tour of a hospital to familiarize children with the environment beforehand. In the study by Carlsson and Henningsson [47], the researchers realized that visiting the operation room might not reduce parents’ or children’s anxiety in surgery care situations. This finding is contradicted by the study of Rantala et al [18], who argued based on a recent meta-analysis that web-based interventions (eg, educational web-based programs or age-appropriate streamed videos) could be used to reduce children’s anxieties [18]. In another study by Rantala et al [23], health specialists observed that a digital gaming solution (developed for a hospital environment, including virtual visits to the hospital for different surgeries) would help families and children to be better oriented to an upcoming treatment. In our study, parents considered a child’s personal involvement in his or her care to be very important. A gaming digital solution developed throughout the care path can solve this challenge. In addition, the World Health Organization [1] raises an important aspect that mHealth could be used to increase patient commitment to their own care and to develop a more personalized path for patient care. In a previous systematic review, gamification was mostly used in chronic diseases and for improving physical activity among patients; there were no designed SHGs for children’s day surgery pathway [4]. Thus, our study produces new knowledge for the rapid day surgery pathway and helps developers consider the parental perspective. In addition, peer support has been used in mHealth app solutions for children with chronic diseases [4,48]. In our study, parents were open to peer support via a gaming digital solution. For future studies, it would be important to examine whether this could be used as an alternative method of patient counseling that could reduce hospital readmissions in children’s day surgeries [14,34].

Overall, the results of the study agree with the existing literature on patients’ expectations and needs related to hip and knee arthroplasty [49]. In this study, the patients suggested that they needed a digital solution that allows for better real-time communication methods (eg, information transfer, discussion forums) and patient counseling (eg, resources, content and implementation). In addition, our results support the previous literature on treatment pathways for surgical patients [49-52]. To the best of our knowledge, prior studies have not focused on the pathways of pediatric day surgeries. In this respect, our study produces new research.

### Conclusion

The parents of children in day surgeries need reliable information about the pathways of those surgeries. Children must be involved in the care paths of their day surgeries. These parents were open to digital gaming solutions. They expressed their thoughts on what kinds of solutions would be relevant to the clinical practice and could positively affect the care provision in hospitals. The digital gaming solution should be developed for the needs of children and provide important information about day surgeries to families. The findings of this study can be applied for integrating digital solutions into hospital environments.

## Appendix

Multimedia Appendix 1 COREQ (Consolidated Criteria for Reporting Qualitative Research) checklist for qualitative research.

## Abbreviations

COREQ: Consolidated Criteria for Reporting Qualitative Research
mHealth: mobile health
MRI: magnetic resonance imaging
PDA: personal digital assistants
QR: quick response
SHG: serious health game
